# Visualization of Middle Ear Ossicles in Elder Subjects with Ultra-short Echo Time MR Imaging

**DOI:** 10.2463/mrms.mp.2015-0171

**Published:** 2016-03-21

**Authors:** Shinji Naganawa, Toshiki Nakane, Hisashi Kawai, Toshiaki Taoka, Kojiro Suzuki, Shingo Iwano, Hiroko Satake, David Grodzki

**Affiliations:** 1 Department of Radiology, Nagoya University Graduate School of Medicine, 65 Tsurumai-cho, Shouwa-ku, Nagoya 466-8550, Japan; 2 Siemens Healthcare GmbH, Magnetic Resonance Imaging

**Keywords:** magnetic resonance imaging, ossicles, temporal bone disease

## Abstract

**Purpose:**

To evaluate the visualization of middle ear ossicles by ultra-short echo time magnetic resonance (MR) imaging at 3T in subjects over 50 years old.

**Materials and Methods:**

Sixty ears from 30 elder patients that underwent surgical or interventional treatment for neurovascular diseases were included (ages: 50–82, median age: 65; 10 men, 20 women). Patients received follow-up MR imaging including routine T_1_- and T_2_-weighted images, time-of-flight MR angiography, and ultra-short echo time imaging (PETRA, pointwise encoding time reduction with radial acquisition). All patients underwent computed tomography (CT) angiography before treatment. Thin-section source CT images were correlated with PETRA images. Scan parameters for PETRA were: TR 3.13, TE 0.07, flip angle 6 degrees, 0.83 × 0.83 × 0.83 mm resolution, 3 min 43 s scan time. Two radiologists retrospectively evaluated the visibility of each ossicular structure as positive or negative using PETRA images. The structures evaluated included the head of the malleus, manubrium of the malleus, body of the incus, long process of the incus, and the stapes. Signal intensity of the ossicles was classified as: between labyrinthine fluid and air, similar to labyrinthine fluid, between labyrinthine fluid and cerebellar parenchyma, or higher than cerebellar parenchyma.

**Results:**

In all ears, the body of the incus was visible. The head of the malleus was visualized in 36/60 ears. The manubrium of the malleus and long process of the incus was visualized in 1/60 and 4/60 ears, respectively. The stapes were not visualized in any ear. Signal intensity of the visible structures was between labyrinthine fluid and air in all ears.

**Conclusion:**

The body of the incus was consistently visualized with intensity between air and labyrinthine fluid on PETRA images in aged subjects. Poor visualization of the manubrium of the malleus, long process of the incus, and the stapes limits clinical significance of middle ear imaging with current PETRA methods.

## Introduction

Imaging studies of middle ear ossicles are usually performed with thin-section computed tomography (CT).^1^ Magnetic resonance (MR) visualization of middle ear ossicles has been reported in normal young adult volunteers using ultra-short echo time (UTE) pulse sequence with radial k-space sampling at 3T with a long scan time of 27 minutes.^2^ Technical developments have allowed further shortening of the echo time of the UTE sequence (i.e., PETRA: pointwise encoding time reduction with radial acquisition).^3^ PETRA allows shorter echo time throughout the k-space than the conventional radial sampling UTE sequence by filling the center of the k-space point by point.^3^ The UTE sequence including PETRA allows quieter scans^4^ and is less susceptible to field inhomogeneity due to air or metallic substances than conventional cartesian spin-echo or gradient echo imaging. UTE sequences are used for imaging the lung,^5^ cortical bone,^6–11^ and vascular structures near metallic substances.^12^ The purpose of this study was to evaluate the visualization of middle ear ossicles in subjects over 50 years old by PETRA at 3T with a shorter scan time.

## Materials and Methods

Sixty ears from 30 elder patients who had undergone surgical or interventional treatment for neurovascular diseases were included (age range: 50–82 years old, median age: 65 years old; 10 men, 20 women). Patients underwent follow-up MR imaging including routine T_1_- and T_2_-weighted images, three-dimensional time-of-flight (3D-TOF) MR angiography (MRA) and UTE MRA using PETRA to evaluate vascular structures, especially those near the metallic coils or the stents. PETRA MRA was performed by subtraction of the image data with an arterial pre-saturation pulse from that without a pre-saturation pulse. All patients had undergone CT angiography before treatment. Thin-section CT source images with a thickness of 0.5–1.0 mm were correlated with source PETRA images obtained without a pre-saturation pulse. The scan parameters for PETRA without a pre-saturation pulse were: repetition time (TR) 3.13 msec, echo time (TE) 0.07 msec, flip angle of 6 degrees, field of view of 213 mm × 213 mm × 213 mm, matrix of 256 × 256 × 256, 0.83 mm × 0.83 mm × 0.83 mm voxels, and scan time of 3 minutes 43 seconds. No pre-inversion pulse or fat suppression pulses were applied. Thus, the contrast of PETRA images in the current study were mildly T_1_-weighted compared to proton density weighted. In only one patient during the initial period of PETRA MRA optimization, PETRA with fat suppression was also obtained.^13^ All MR imaging was performed at 3T (Magnetom Skyra, Erlangen, Germany) using a 32-channel array head coil. Two radiologists retrospectively evaluated the visibility of each middle ear ossicle structure as either positive or negative based on PETRA images in reference to the CT images. If there were any discrepancies between the two evaluators, a consensus was obtained after discussion. The ossicular structures evaluated were the head of the malleus, manubrium of the malleus, body of the incus, long process of the incus, and the stapes. The signal intensity of the visualized structures were classified as one of the following: (1) between labyrinthine fluid and air, (2) similar to labyrinthine fluid, (3) between labyrinthine fluid and cerebellar parenchyma, or (4) higher than cerebellar parenchyma. The location of metallic objects and the presence of metallic effects on the middle ear on PETRA were recorded. The medical ethics committee of our institution approved this retrospective study with a waiver of written informed consent from the patients.

## Results

**Fig 1.**
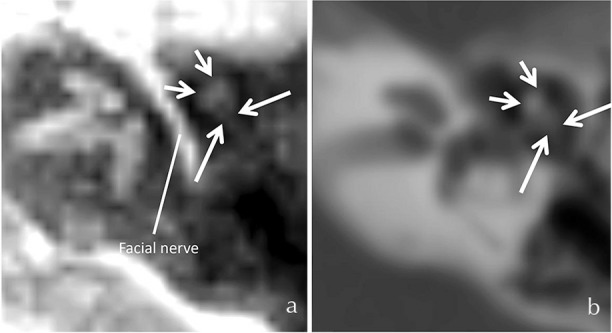
A 64-year-old woman. The head of the malleus (short arrows) and body of the incus (long arrows) in the left middle ear are visualized with a signal intensity between labyrinthine fluid and air on this PETRA image (**a**) which corresponds to the computed tomography image (**b**). Note that the facial nerve shows a higher signal than the labyrinthine fluid (**a**). PETRA, pointwise encoding time reduction with radial acquisition.

**Fig 2.**
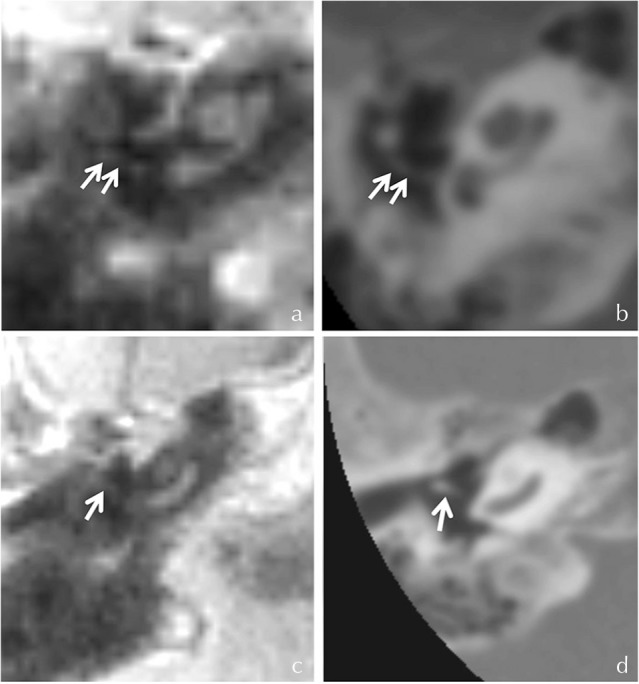
A 74-year-old woman. The long process of the incus (arrows) in the right middle ear is faintly visualized with a signal intensity between labyrinthine fluid and air on this PETRA image (**a**) which corresponds to the CT image (**b**). The manubrium of the malleus (arrow) in the right middle ear is faintly visualized with a signal intensity between labyrinthine fluid and air on this PETRA image (**c**) which corresponds to the CT image (**d**). CT, computed tomography; PETRA, pointwise encoding time reduction with radial acquisition.

In all 60 ears, the body of the incus could be visualized. The head of the malleus was visible in 36 ears out of 60 ears. The manubrium of the malleus and long process of the incus was visualized only occasionally, in 1/60 and 4/60 ears, respectively (Figs. 1, 2). The stapes could not be visualized in any ear. The signal intensity of the visible structures of the ossicles was between labyrinthine fluid and air in all ears. No patient had otitis media or ossicular anomalies based on the CT images.

**Fig 3.**
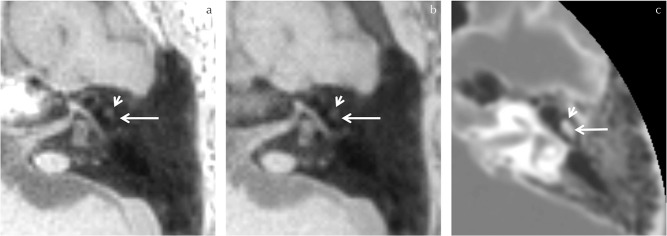
A 64-year-old woman. PETRA image without fat suppression pulses (**a**), PETRA image with fat suppression pulses (**b**) and the corresponding CT image (**c**). The head of the malleus (short arrow in each image) and the body of the incus (long arrow in each image) are similarly visualized by PETRA with and without fat suppression pulses. The signal of the ossicles appears to come not from fat, but from water. CT, computed tomography; PETRA, pointwise encoding time reduction with radial acquisition.

In one subject who underwent PETRA both with and without fat suppression pulses, the ossicles showed similar visibility on both types of images (Fig. 3). The details of the PETRA visualization for each structure of the middle ear ossicles are summarized in Table 1. On the PETRA images, no apparent metallic artifacts were noted in the vicinity of the middle ear in any patient. The location of the metallic substances included 2 in the anterior communicating artery, 11 in the internal carotid artery, 7 in the middle cerebral artery, 2 in the posterior cerebral artery, 3 in the basilar artery, 4 in the vertebral artery, and 1 in the cavernous sinus.

## Discussion

Prior to the development of the UTE sequence, middle ear ossicles could only be recognized as a signal void when the surrounding middle ear cavity was occupied by fluid or tissue. The MR signals of middle ear ossicles themselves can now be obtained using the UTE sequence. In the present study, ossicular signals could be obtained even with fat suppression pulses. This suggests that the signal from the middle ear ossicles likely come from water, not from fat.

In the present study, the body of the incus was visualized in all ears. However, the head of the malleus was visible in only 60% of the ears. The cause of this difference is unknown, but we speculate that this is likely due to the smaller sized head of the malleus compared to the body of the incus. In many ears, the head of the malleus showed visibly reduced signals compared to the body of the incus. However, in some ears, the head of the malleus showed slightly higher signals than the body of the incus. The variation in water content between the malleus and incus might influence their visualization, in addition to the size difference between the two structures.

Water is present in cortical bone in different bound states.^14^ Cortical bone consists of minerals (<43%), organic matrix (<35%), and water (<22%). Free water, which constitutes a small fraction of the total water, is present in the haversian canals, lacunae, and canaliculi. Bound water, which makes up the larger portion of water, is found in crystals of apatite-like minerals and the organic matrix. UTE is effective for visualizing bound water.^10^ Bound and free (or pore) water can be visually separated by selecting an inversion time which nullifies the pore water in cortical bone.^14^ UTE MR imaging allows for the assessment of cortical bone by detection of proton signals from mobile water in the pore spaces (i.e., pore water) and water bound to the collagen matrix by hydrogen bonds (i.e., bound water).^15^ Bound and pore water can also be visually separated by comparing the signal at short and long TE, because bound water has very short T_2_ values.

Cortical porosity is a major determinant of overall bone strength.^15^ An indicator of cortical bone porosity, called the porosity index, is defined as the ratio of UTE image intensities at a long and short TE, and the results can be compared with biexponential analysis.^15^ In the present study, we obtained only short TE images, but it is possible that we could also obtain longer TE images simultaneously with slight scan time elongation.

The invasion of cholesteatomas into the middle ear ossicles could alter ossicular porosity. Thus further study is warranted to determine if porosity might be an earlier marker for cholesteatoma invasion into the ossicles than morphological changes detectable on CT. In the present state, CT would remain the modality of choice for the assessment of middle ear disease.

The calcium and phosphorus content of the mallei in both men and women have been shown to remain constant throughout ages ranging from 40–98 years.^16^ It has been also reported that there was no significant difference in mineral content between men and women.^17^

A previous study employed a TE of 0.14 ms which represented a shorter echo time. The echo time of 0.14 ms was double the time used in the present study. In that study, young adult volunteers were scanned with a 27-minute scan time.^2^ The ossicles appeared as a complex of the malleus and incus at the incudomalleolar joint.^2^ They did not evaluate each ossicle type and their structures separately. In the present study, we scanned elder subjects in 3 minutes 43 seconds and evaluated the visualization of each ossicular structure separately. The shorter scan time and the individual evaluation of each structure might make the present study more feasible in a clinical setting than the previous study. The results of the present study showed effective visualization of ossicles even in elder subjects. Although clear aging effects in the middle ear ossicles has not been reported, confirmation of ossicle visualization in elder subjects by MR imaging warrants further research with a population of a more varied age range.

The PETRA slice thickness used in this study was 0.83 mm. Current high resolution CT employs a 0.5 mm thickness; however ultra-high-resolution CT with 0.25 mm thickness is expected to be available in clinical practice in the near future.^18^ The evaluation of middle ear ossicles by PETRA imaging will depend on higher spatial resolution to be of value in the clinical field. PETRA images in the present study are mild T_1_-weighted images, which might be the supplemental images rather than the replacement of routine T_1_-weighted images.

There are several limitations in the present study. The retrospective nature of this study allowed some selection bias. All subjects had been treated for various cerebrovascular diseases. No patient had otitis media. Therefore, visibility of ossicles in the patients with otitis media is unknown. Reference CT images were obtained as the source image for CT angiography, therefore CT images were not optimized for the evaluation of ossicles. Although the morphology of the ossicles could still be determined on the source image for CT angiography, further study including the comparison with dedicated middle ear CT images should be conducted in the future. Furthermore, PETRA images are optimized for MR angiography not for the visualization of the ossicles. Further improvements for middle ear ossicles might be possible. Evaluation of PETRA images was subjective due to the small size of the ossicles. Although some structures of the ossicles (i.e., the body of the incus) could be visualized in all ears, the manubrium of the malleus and long process of the incus were only occasionally visible. Even the head of the malleus was visualized in only 60% of the ears, and the stapes were not visualized in any ear. Poor visualization of the small but important ossicular structures limits the clinical significance of middle ear imaging by current forms of PETRA. Further improvements in spatial resolution and contrast will likely be necessary to expand the utility of PETRA as a clinical evaluation tool for middle ear ossicles.

## Conclusion

The body of the incus was consistently visualized with a signal intensity between air and labyrinthine fluid on PETRA even in aged subjects. Poor visualization of smaller structures such as the manubrium of the malleus, the long process of the incus, and the stapes needs to be improved to apply MR imaging-based evaluation of middle ear ossicles in a clinical setting.

## Figures and Tables

**Table 1.** Number of ears (out of 60) with specific ossicular structures visible on PETRA

Head of malleus	Manubrium of malleus	Body of incus	Long process of incus	Stapes
36/60	1/60	60/60	4/60	0/60
